# Conditions for excellence in teaching in medical education: The Frankfurt Model to ensure quality in teaching and learning

**DOI:** 10.3205/zma001123

**Published:** 2017-10-16

**Authors:** Marianne Giesler, Gudrun Karsten, Falk Ochsendorf, Jan Breckwoldt

**Affiliations:** 1 Albert-Ludwigs-Universität Freiburg, Med. Fakultät, Studiendekanat, Kompetenzzentrum Evaluation in der Medizin Baden-Württemberg, Freiburg, Germany; 2 Christian-Albrechts-Universität zu Kiel, Medizinische Fakultät, Dekanat, KiMed Zentrum für Medizindidaktik, Kiel, Germany; 3 J. W. Goethe-Universität Frankfurt/M, Universitätsklinikum, Klinik für Dermatologie, Venerologie und Allergologie, Frankfurt/M., Germany; 4 Universität Zürich, Medizinische Fakultät, Dekanat, Stab Forschung und Entwicklung, Zürich, Switzerland

**Keywords:** Evaluation, Quality of Teaching and Learning, Teaching and Learning conditions

## Abstract

**Background: **There is general consensus that the organizational and administrative aspects of academic study programs exert an important influence on teaching and learning. Despite this, no comprehensive framework currently exists to describe the conditions that affect the quality of teaching and learning in medical education. The aim of this paper is to systematically and comprehensively identify these factors to offer academic administrators and decision makers interested in improving teaching a theory-based and, to an extent, empirically founded framework on the basis of which improvements in teaching quality can be identified and implemented.

**Method: **Primarily, the issue was addressed by combining a theory-driven deductive approach with an experience based, “best evidence” one during the course of two workshops held by the GMA Committee on Personnel and Organizational Development in Academic Teaching (POiL) in Munich (2013) and Frankfurt (2014). Two models describing the conditions relevant to teaching and learning (Euler/Hahn and Rindermann) were critically appraised and synthesized into a new third model. Practical examples of teaching strategies that promote or hinder learning were compiled and added to the categories of this model and, to the extent possible, supported with empirical evidence.

Based on this, a checklist with recommendations for optimizing general academic conditions was formulated.

**Results: **The *Frankfurt Model of conditions to ensure Quality in Teaching and Learning* covers six categories: *organizational structure/medical school culture, regulatory frameworks, curricular requirements, time constraints, material and personnel resources, *and *qualification of teaching staff.* These categories have been supplemented by the interests, motives and abilities of the actual teachers and students in this particular setting. The categories of this model provide the structure for a checklist in which recommendations for optimizing teaching are given.

**Conclusions: **The checklist derived from the Frankfurt Model for ensuring quality in teaching and learning can be used for quality assurance and to improve the conditions under which teaching and learning take place in medical schools.

## Zusammenfassung

**Hintergrund: **Es gilt allgemein als akzeptiert, dass Rahmenbedingungen einen wichtigen Einfluss auf Lehren und Lernen ausüben. Trotzdem existiert für das Studium der Medizin bislang kein umfassendes Rahmenwerk zur Beschreibung von Bedingungen, die Einfluss auf die Lehr-/Lernqualität haben. Ziel der vorliegenden Arbeit war es daher, diese Faktoren systematisch und umfassend zusammenzustellen, um Fakultätsleitungen und allen an der Verbesserung der Lehre interessierten Entscheidungsträgern ein theoriegeleitetes und - in Ansätzen - empirisch fundiertes Rahmenwerk anzubieten, mit dessen Hilfe Maßnahmen zur Optimierung der Lehrqualität begründet und umgesetzt werden können.

**Vorgehen:** In zwei Workshops des GMA-Ausschusses Personal und Organisationsentwicklung in der Lehre (POiL) in München (2013) und Frankfurt (2014) wurde die Thematik zunächst theorie-, dann erfahrungsgeleitet bearbeitet. Zwei Modelle zur Beschreibung von lehr-/lernrelevanten Rahmenbedingungen (von Euler/Hahn und Rindermann) wurden kritisch diskutiert und zu einem neuen Modell zusammengeführt. Zu den Kategorien dieses Modells wurden lernförderliche und -hinderliche Praxisbeispiele zusammengetragen und – soweit möglich – mit empirischen Belegen gestützt. Basierend auf dieser Arbeit wurde eine Checkliste mit Empfehlungen zur Optimierung der Rahmenbedingungen formuliert.

**Ergebnisse: **Das* Frankfurter Modell der Rahmenbedingungen zur Sicherung der Lehr- und Lernqualität* umfasst die sechs Kategorien: *Organisationsstruktur/Kultur der Fakultät, rechtliche Bedingungen, curriculare Vorgaben, zeitliche Bedingungen, materielle* *und Personalausstattung* sowie *Qualifizierung des Lehrpersonals*. Ergänzt werden diese Kategorien durch die in diesem Kontext agierenden Lehrenden und Studierenden mit ihren Interessen, Motiven und Fähigkeiten. Die Kategorien dieses Modells bilden die Struktur für eine Checkliste, in der Empfehlungen zur Optimierung der Lehre aufgeführt werden.

**Schlussfolgerungen: **Die vom Frankfurter Modell der Rahmenbedingungen zur Sicherung der Lehr- und Lernqualität abgeleitete Checkliste kann zur Qualitätssicherung und Optimierung der der Lehr-/Lernbedingung an medizinischen Fakultäten eingesetzt werden.

## Background

Curriculum designers, department heads and policy makers have many options regarding medical education, some of which they are very possible unaware. Knowledge of general organizational and administrative aspects could help in the selection of targeted and effective interventions. Accordingly, the German Council of Science and Humanities lists numerous aspects pertaining to teaching in its 2012 guideline for evaluating university medical schools and institutions [1]. The main categories are *structure and organization of the academic study program, professionalism of the teaching, quality assurance of the teaching, and teaching infrastructure.* The World Federation for Medical Education (WFME) [2] views the learning environment as significant for the evaluation of medical programs. In the *Charta guter Lehre* [https://www.stifterverband.org/charta-guter-lehre], drafted under the encouragement of the *Stifterverband für die Deutsche Wissenschaft,* there are extensive recommendations to create conditions for excellence in higher education. This document contains many best-practice examples, but provides no concrete references to medical education. Such connections are of great importance when meeting the challenges faced in university medicine, for instance, the rivalry between academic teaching and providing medical care, the high financial costs associated with the studies of medicine, the necessity for strictly structured curricula, and the great demands placed by society on medical graduates (reliability, patient safety, etc.).

For all of the works cited above there is no comprehensive system that shows how these organizational aspects work or can be controlled. It appears particularly important to consider the different degrees to which conditions can be shaped or influenced to optimally use any potential flexibility.

Two models are of help when classifying quality-influencing conditions: Euler and Hahn’s [3] model describing the conditions for teaching and the quality assurance model proposed by Donabedian [4], which was originally developed for the healthcare sector. The first model identifies six categories of university-relevant conditions, described below. Donabedian’s model encompasses the quality dimensions of structure, process and outcome, and has the advantage of describing variables that are relatively concrete and verifiable. Its disadvantage is that the correlations between structure, process, and outcome are not considered. Furthermore, it is without question that in educational settings the interactions between those directly involved in the teaching and learning processes must be included. These participants include students with their different interests, motives and varying degrees of prior knowledge, as well as instructors with their varying methods and teaching abilities. The complex interaction between them and the setting can be captured well by situativity theory [5] and other similar approaches [6], [7], [8] since relevant complex (nonlinear and multiphase) interactions in a situation are emphasized. Of importance for higher education is the multidimensional model of successful teaching put forth by Rindermann [9] whose elements can be assigned to the dimensions identified by Donabedian and even show the relationships and interactions between these elements.

Curriculum designers, department heads and key policy makers carry a social responsibility for educational programs [10], [11]. This circle of people should therefore be familiar with the basic conditions that promote teaching and learning, primarily given that in the case of medicine there is, in addition to the classic academic rivalry between teaching and research, serious competition with the provision of medial care [12].

The aim of this paper is to develop a model for medical education with which the relevant organizational and administrative conditions related to teaching and learning can be described within the context of increasing healthcare demands. Based on this model, recommendations are intended for effective and efficient creation of conditions conducive to teaching and learning.

## Methods

A Working Group of the German Association for Medical Education’s Committee on Personnel and Organizational Development [https://gesellschaft-medizinische-ausbildung.org/aktivitaeten/ausschuesse/personal-und-organisationsentwicklung/mitglieder.html held two workshops in 2013 and 2014 in Munich and Frankfurt to address the issue of favorable and unfavorable conditions for teaching and learning. The workshops were conducted in the form of moderated discussions. To provide structure following intensive debate, only the two models best suited to medical education were drawn upon and applied.

All of the participants had many years of experience in medical education and had completed either advanced post-licensure training in psychology, or medical education and medicine. Prior to the first workshop the participants filled out a matrix regarding the organizational aspects and general conditions at their own medical schools which foster or impede learning. This matrix was based on the model by Euler and Hahn [3] and covered six categories of general conditions relevant to higher education (see below). The positive and negative conditions based on experience were compiled in the first workshop and discussed. Potential problems and solutions were then identified. Since it became clear during the first workshop that Euler and Hahn’s model did not establish connections to teaching, Rindermann’s multidimensional model for teaching outcomes [9] was integrated into Euler and Hahn’s model during the second workshop. As a result, the interactions of the people participating in the teaching and learning processes and their relationship to the outcome were taken into account. Subsequently, empirically-based approaches were sought for the positive and negative conditions. The results of this paper form the basis for a checklist to facilitate quality assurance and the optimization of teaching at medical schools.

## The Frankfurt Model of conditions to ensure the quality of teaching and learning

**Figure 1:**
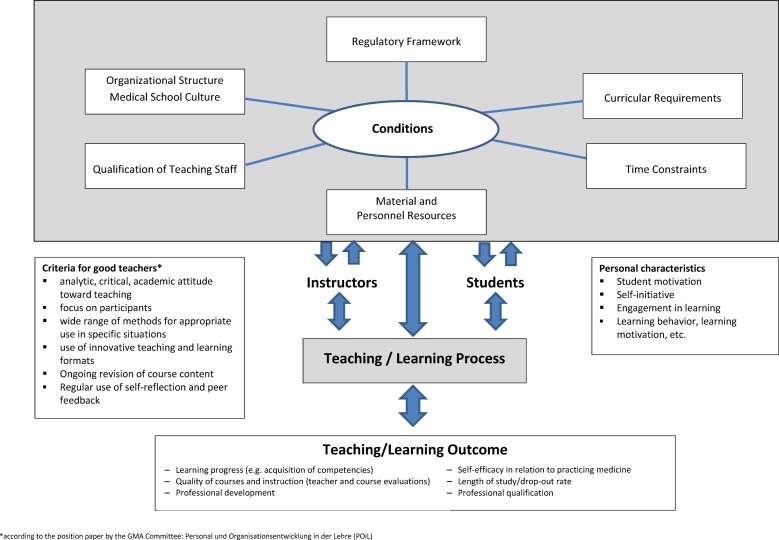
The Frankfurt Model of conditions to ensure the quality of teaching and learning

The Frankfurt Model^1^ of conditions to ensure the quality of teaching and learning (see figure 1 (Fig. 1)) synthesizes two already established models. The categories in the model described by Euler und Hahn [3] can be described as follows:

1. *Organizational structure/medical school culture*: regulations, rules and measures can, among other things, steer department procedures (e.g. general philosophy, organigrams) [13]. This also applies to the commonly shared unwritten assumptions, values and expectations (culture) of those on the faculty and staff. Based on this, many different interventional measures to optimize teaching can present themselves.
2. *Regulatory frameworks:* here it must be ascertained whether there is room to maneuver within the existing framework and how this flexibility can be used most meaningfully. For example, the use of pilot study program clauses and the introduction of controls to stop any additional applicants from suing for admission fall under this category.
3. With *curricular requirements* curriculum designers have the greatest opportunity to set a course of direction or initiate a change. They can, for instance, ensure that the curricular content in natural sciences and the clinical subjects is vertically integrated. Further opportunities exist in that a specific educational philosophy (e.g. POL) becomes anchored in the medical school or that focus is placed on a specific area (e.g. general practice).
4. *Qualification of teaching staff:* the quality of teaching is a critical factor in an educational setting [14], [15]. Since the balance between teaching and other responsibilities can vary dramatically among instructors, medical schools have the challenge of pragmatically “dosing out” qualification measures as they are needed.
5. The category* material and personnel* resources encompasses classroom spaces and teaching materials (e.g. devices for practical tutorials and simulators), audio/visuals for the various teaching and learning methods, and the availability of sufficiently qualified staff for planning, organization and implementation. In terms of materials, the critical question must be asked if new acquisitions always lead to better educational outcomes [16], [17].
6. The final category of Euler and Hahn’s model describes *time constraints*. This includes not only the time instructors spend in the classroom, but also the time available to prepare and evaluate class sessions and to develop teaching. For students this means the time needed to prepare for and follow-up on class sessions and the time spent traveling to and from class.

In order to include the actors and the teaching/learning process and the outcome of this process, a second model, Rindermann’s multidimensional model of successful teaching [9], was selected and integrated into the first model. According to this model an optimal outcome is the goal of academic teaching – despite all the difficulties of quantifying it. Measurable outcomes, such as exam scores, do not automatically allow for conclusions to be drawn about teaching quality in a particular subject [18], [19], [20], [21]. Other indicators of good teaching such as raising interest in the learning material, strengthening self-efficacy, and a professionalization of the student are also not simple to measure or analyze. For instance, at the beginning of their studies students already possess knowledge about different areas of interest that influence their learning behavior [22]. In addition, their skills, motivation and attitudes toward the program and its organizational and administrative aspects can be influenced [23], [24], [25]. Note must also be taken of conditions that are not connected with the teaching/learning process at hand or that are not within the instructor’s control (e.g. fairness variables, such as heat, overfull classes, acoustics [9], [26]).

## Checklist of conditions conducive to teaching and learning

Recommendations for medical schools were articulated by the Working Group based on the Frankfurt Model (above) detailing the conditions conducive to teaching and learning. These recommendations are supported by the empirical evidence available at this time and are listed in the form of a checklist in attachment 1 .

The recommendations contained in the checklist are not based on systematic literature research. This checklist should therefore be gradually expanded and updated.

## Conclusion

The organizational and administrative aspects of academic study programs exert significant influence on teaching and learning. For this reason decision makers at medical schools need to know which conditions are relevant and how they can be optimally shaped or influenced to improve teaching. With the Frankfurt Model’s ability to identify program aspects that foster quality in teaching and learning, a model has been developed that captures important university-relevant conditions [1], [2], [3], [4] and also incorporates the complexity of the teaching and learning process [5], [8]. Based on this model a checklist of recommendations for good practice has been drafted to enable medical schools to analyze and optimize, where needed, the conditions under which teaching and learning take place at their institutions.

This paper also offers a theoretical foundation for recommendations to ensure quality in teaching and learning at medical schools.

## Notes

^1^One of the two workshops, along with a subsequent meeting, took place in Frankfurt under wonderfully hospitable circumstances making this designation seem appropriate to us.

## Competing interests

The authors declare that they have no competing interests. 

## Supplementary Material

Checklist: Recommendations for creating conditions for excellence in teaching in medical education

## References

[1] Wissenschaftsrat (2012). Leitfaden der Evaluation universitätsmedizinischer Einrichtungen.

[2] Federation for Medical Education (1998). International Standards in medical education: assessment and accreditation of medical schools'programmes: A WFME position paper. Med Educ.

[3] Johansen K (2010). Einsteigerhandbuch Hochschullehre. Aus der Praxis für die Praxis.

[4] Donabedian A (2005). Evaluating the Quality of Medical Care. Milbank Q.

[5] Endler NS, Edwards JM (1986). Interactionism in personality in the twentieth century. Person Individ Diff.

[6] Frederiksen N (1972). Toward a taxonomy of situations. Am Psychol.

[7] Lantermann ED (1980). Interaktionen: person, Situation und Handlung.

[8] Durning SJ, Artino AR (2011). Situativity theory: A perspective on how participants and the environment can interact: AMEE Guide no. 52. Med Teach.

[9] Rindermann H (2001). Lehrevaluationen - Einführung und Überblick zu Forschung und Praxis der Lehrveranstaltungsevaluation an Hochschulen. Mit einem Beitrag zur Evaluation computerbasierten Unterrichts.

[10] Schimank U, Altrichter H, Brüsemeister T, Wissinger J (2007). Die Governance-Perspektive: Analytisches Potenzial und anstehende konzeptionelle Fragen. Educational Governance – Handlungskoordination und Steuerung im Bildungssystem.

[11] Becker FG, Becker FG, Krücken G, Wild E (2012). Governance von Hochschulen: Einfluss von organisatorischen Rahmenbedingungen auf "gute Lehre". Gute Lehre in der Hochschule: Wirkungen von Anreizen, Kontextbedingungen und Reformen.

[12] Albanese M, Mejicano G, Gruppen L (2008). Competency-based medical education: A defense against the four horsemen of the medical education Apocalypse. Acad Med.

[13] Weinert AB (1998). Organisationspsychologie.

[14] Steinert Y, Mann K, Centeno A, Dolmans D, Spencer J, Gelula M, Prodeaus D (2006). A systematic review of faculty development initiatives designed to improve teaching effectiveness in medical education: BEME guide No. 8. Med Teach.

[15] Hattie J (2009). Visible learning: A synthesis of over 800 meta-analyses relating to achievement.

[16] Issenberg SB, McGaghie WC, Petrusa ER, Gordon DL, Scalese RJ (2005). Features and uses of high-fidelity medical simulations that lead to effective learning: a BEME systematic review. Med Teach.

[17] McGaghie WC, Issenberg SB, Petrusa ER, Scalese RJ (2010). A critical review of simulation-based medical education research: 2003–2009. Med Educ.

[18] Biller S, Boeker M, Fabry F, Giesler M (2015). Impact of the Medical Faculty on Study Success in Freiburg: Results from Graduate Surveys. GMS Z Med Ausbild.

[19] Greb AE, Brennan S, McParlane L, Page R, Bridge PD (2009). Retention of medical genetics knowledge and skills by medical students. Genet Med.

[20] Ramchandani D (2011l). Grading medical students in a psychiatry clerkship: correlation with the NBME subject examination scores and its implications. Acad Psych.

[21] Hamdy H, Prasad K, Anderson MB, Scherpbier A, Williams R, Zwierstra R, Cuddihy H (2006). BEME systematic review: predictive values of measurements obtained in medical schools and future performance in medical practice. Med Teach.

[22] Krapp A, Achtenhagen F, Lempert W (2000). Individuelle Interessen als Bedingung lebenslangen Lernens. Lebenslanges Lernen im Beruf - seine Grundlegung im Kindes- und Jugendalter. Band 3: Psychologische Theorie, Empirie und Therapie.

[23] Fabry G, Giesler M (2007). Hochmotiviert am Start: Zur Studienmotivation von Medizinstudenten während des ersten Studienjahres. Z Med Psychol.

[24] Metzger C, Schulmeister R, Martens T (2012). Motivation und Lehrorganisation als Elemente von Lernkultur. Z Hochschulentwickl.

[25] Winteler A, Forster P (2008). Lern-Engagement der Studierenden – Indikator für die Qualität und Effektivität von Lehre und Studium. Hochschulwes.

[26] Rindermann H (1996). Untersuchungen zur Brauchbarkeit studentischer Lehrveranstaltungen.

[27] Hafler JP, Ownby AR, Thompson BM, Fasser CE, Grigsby K, Haidet P, Kahn MJ, Hefferty FW (2011). Decoding the Learning Environment of Medical Education: A Hidden Curriculum Perspective for Faculty Development. Acad Med.

[28] Genn JM (2001). AMEE Medical Education Guide No. 23 (Part 1): Curriculum, environment, climate, quality and change in medical education—a unifying perspective. Med Teach.

[29] Genn JM (2001). AMEE Medical Education Guide No. 23 (Part 2): Curriculum, environment, climate, quality and change in medical education – a unifying perspective. Med Teach.

[30] Reich, K (2006). Was ist eine gute Lernumgebung?. Konstruktivistische Didaktik.

[31] Bland CJ, Starnaman S, Wersal L, Moorhead-Rosenberg L, Zonia S, Henry R (2000). Curricular Change in Medical Schools: How to Succeed. Acad Med.

[32] Becker FG (2012). Motivation und Anreize "zu guter Lehre" von Neuberufenen. Schlussbericht der deutschlandweiten Befragung von Neuberufenen Professor(inn)en im Rahmen des BMBF-geförderten MogLi-Projekts.

[33] Kuhnigk O, Schreier J, Harendza S (2013). Sustained change in didactic skills – does teacher training last? GMS Z Med Ausbild. http://dx.doi.org/10.3205/zma000880.

[34] Sim JH, Tong WT, Vadivelu J, Hassan H (2015). Development of an instrument to measure medical students' perceptions of the assessment environment: Initial validation. Med Educ Online.

[35] Rotthoff T, Ostapczuk MS, De Bruin J, Decking U, Schneider M, Ritz-Timme S (2011). Assessing the learning environment of a faculty: Psychometric validation of the German version of the Dundee Ready Education Environment Measure with students and teachers. Med Teach.

[36] Strand P, Sjöborg K, Stalmeijer R, Wichmann-Hansen G, Jakobsson U, Edgren G (2013). Development and psychometric evaluation of the Undergraduate Clinical Education Environment Measure (UCEEM). Med Teach.

[37] Boor K, van der Vleuten C, Teunissen P, Scherpbier A, Scheele F (2011). Development and analysis of D-RECT, an instrument measuring residents' learning climate. Med Teach.

[38] Wissenschaftsrat (2014). Empfehlungen zur Weiterentwicklung des Medizinstudiums in Deutschland auf Grundlage einer Bestandsaufnahme der humanmedizinischen Modellstudiengänge.

[39] Bremer C (2011). E-Learning als Innovation in der Lehre – Ansätze zur hochschulweiten Organisationsentwicklung. ZFHE.

[40] Ten Cate O, Chen HC, Hoff RG, Peters H, Bok H, van der Schaaf M (2015). Curriculum development for the workplace using Entrustable Professional Activities (EPAs): AMEE Guide No. 99. Med Teach.

[41] Biggs B (1996). Enhancing teaching through constructive alignment. High Educ.

[42] Weinert FE (1982). Selbstgesteuertes Lernen als Voraussetzung, Methode und Ziel des Unterrichts. Unterrichtswissenschaft.

[43] Sobral DT (2000). Medical students' reflection in learning in relation to approaches to study and academic achievement. Med Educ.

[44] Kern DE, Thomas PA, Howard SM, Bass EB (1998). Curriculum development for medical education – a six-step approach.

[45] Breckwoldt J, Peters H, Behrendt B (2012). Modellcurriculum in der medizinischen Ausbildung: Das Beispiel Charité. Neues Handbuch Hochschullehre: Lehren und Lernen effizient gestalten.

[46] Jünger J, Just I (2014). Empfehlungen der Gesellschaft für Medizinische Ausbildung und des Medizinischen Fakultätentags für fakultätsinterne Leistungsnachweise während des Studien der Human-, Zahn- und Tiermedizin. GMS Z Med Ausbild.

[47] Nouns ZM, Georg W (2010). Progress testing in German speaking countries. Med Teach.

[48] Wrigley W, van der Vleuten CP, Freeman A, Muijtjens A (2012). A systemic framework for the progress test: strengths, constraints and issues: AMEE Guide No. 71. Med Teach.

[49] Ross MT, Cameron HS (2007). Peer assisted learning: A planning and implementation framework: AMEE Guide no. 30. Med Teach.

[50] Blohm M, Lauter J, Branchereau S, Krautter M, Köhl-Hackert N, Jünger J, Herzog W, Nickendei C (2015). Peer-Assisted Learning" PAL im Skills-Lab – eine Bestandsaufnahme an den Medizinischen Fakultäten der Bundesrepublik Deutschland. GMS Z Med Ausbild.

[51] Taherian K, Shekarchian M (2008). Mentoring for doctors. Do its benefits outweigh its disadvantages?. Med Teach.

[52] Belcher R, Jones A, Smith LJ, Vincent T, Naidu S, Montgomery J, Haq I, Gill D (2014). Qualitative study of the impact of an authentic electronic portfolio in undergraduate medical education. BMC Med Educ.

[53] Liebhardt H, Fegert JM, Dittrich W, Nürnberger F (2010). Medizin studieren mit Kind – ein Trend der Zukunft?. Dtsch Arztebl.

[54] Binninger S, Brüstle P, Korinthenberg R, Streitlein-Böhme I (2012). Förderung der Familienfreundlichkeit an der Medizinischen Fakultät Freiburg – Bilanzen der Studienteilnahme. GMS Z Med Ausbild.

[55] Brüstle P, Biller S, Giesler M (2011). Studien- und Lebenssituation von Medizinstudierenden an der Universität Freiburg. ZFHE.

[56] Johannes C, Seidel T (2012). Professionalisierung von Hochschullehrenden. Lehrbezogene Vorstellungen, Wissensanwendung und Identitätsentwicklung in einem videobasierten Qualifikationsprogramm. Z. Erziehungswiss.

[57] Breckwoldt J, Svensson J, Lingemann C, Gruber H (2014). Does clinical teacher training always improve teaching effectiveness as opposed to no teacher training? A randomized controlled study. BMC Med Educ.

[58] Lammerding-Köppel M, Fabry G, Hofer M, Ochsendorf F, Schirlo C (2006). Hochschuldidaktische Qualifizierung in der Medizin: I. Bestandsaufnahme. GMS Z Med Ausbild.

[59] Lammerding-Köppel M, Fabry G, Hofer M, Ochsendorf F, Schirlo C (2006). Hochschuldidaktische Qualifizierung in der Medizin: II. Anforderungsprofil der Qualifizierungsangebote. GMS Z Med Ausbild.

[60] Steinert Y, Mann K, Centeno A, Dolmans D, Spencer J, Gelula M, Prodeaus D (2006). A systematic review of faculty development initiatives designed to improve teaching effectiveness in medical education: BEME guide No. 8. Med Teach.

[61] Steinert Y, Macdonald ME, Boillat M, Elizov M, Meterissian S, Razack S, Ouellet MN, McLeod PJ (2010). Faculty development: if you build it, they will come. Med Educ.

[62] Nordquist J, Sundberg K, Laing A (2016). Aligning physical learning spaces with the curriculum: AMEE Guide No. 107. Med Teach.

[63] Nelson C, Hartling L, Campbell S, Oswald AE (2012). The effects of audience response systems on learning outcomes in health professions education. A BEME systematic review: BEME Guide No. 21. Med Teach.

[64] Westermann J, Brauner A (2011). Medizinstudium: "Gefühlte" Belastung als Parameter für die Organisation eines erfolgreichen Curriculums. Dtsch Med Wochenschr.

[65] Schmidt HG, Cohen-Schotanus J, van der Molen HT, Splinter TA, Bulte J, Holdrinet R, van Rossum HJ (2010). Learning more by being taught less: a "time-for-self-study" theory explaining curricular effects on graduation rate and study duration. High Educ.

