# Supplementary material for: Conditions for excellence in teaching in medical education: The Frankfurt Model to ensure quality in teaching and learning
Source: GMS J Med Educ. 2017 Oct 16;34(4):Doc46. doi: 10.3205/zma001123 (PMC5654116; doi:10.3205/zma001123)
Supplement: Checklist: Recommendations for creating conditions for excellence in teaching in medical education [file JME-34-46-s-001.pdf]

**Organizational Structure / Medical School Culture** [1], [9], [23–35]	**exists**
**Clear and public commitment of the medical school** to teaching<br>*Characteristics / Actions:*	
- Philosophy of teaching	
- Visualization of the people responsible for academic teaching (organigram)	
- Openly accessible information on times, rooms, teaching formats, learning objectives (online platform)	
- Ongoing, confidence-building work of the executive committees	
- Showcasing of academic teaching (e.g. specific days dedicated to topics related to teaching)	
- Transparent and separate accounting for teaching and research	
- Teaching professorships / *Tenure track*	
**Teacher Support**<br>*Characteristics / Actions:*	
- Structured mandatory incoming teacher training program (Inplacement)	
- Anchoring of teaching duties into faculty schedules	
- Teacher training and development programs	
- Individual incentive programs and appreciation for good teaching (Teacher of the Month/Year)	
- Management through institutionalized incentive programs	
**Systematic Analysis of Quality of Teaching and Learning** (curriculum, module, and class session)<br>*Characteristics / Actions*:	
- Use of valid measuring instruments	
- Use of measuring criteria relevant to common clinical settings	
**Educational Policy**<br>*Characteristics / Actions:*	
- Advancement of teaching on the level of (educational) policy making	
- Separate allocation of budgets for teaching and research	

**Regulatory Framework**	**exists**
Characteristics / Actions:	
- Implementation of the legal frameworks for the purpose of:<br>- consistency using any flexibility to the advantage of teaching<br>- complying with legal requirements (e.g. group sizes)	
**Drafting rules and regulations at the level of the university and medical school** (study program, examinations, admission requirements)	
**Influence** (if possible) **regarding legal requirements and statutes at the state and national level:**	
- **Germany**: Regulations governing medical licensure (ÄAppO), Regulations governing student admissions (KapVO), Framework act for higher education (HRG), State laws governing universities and higher education (LHG)	
- **Austria**: University Act of 2002 (UG 2002), the rules and regulations of the specific universities	
- **Switzerland**: Act governing medical professions (MedBG), Cantonal rules for restricting admissions	
- Rules and regulations of the European Union [(http://eur-lex.europa.eu/legal-content/DE/TXT/?uri=celex:32013L0055)	
**Curricular Requirements** (explicit in curriculum) [36–56]	**exists**
**Curricula based on Learning Theory** [http://www.ibe.unesco.org/publications/educationalpracticesseriespdf/prac01e.pdf]<br>*Characteristics / Actions:*	
- Curriculum with a consistent structure in alignment with the educational philosophy and profile of the medical school	
- Learning objectives matched to expected outcomes (e.g. competencies or EPAs (entrustable professional activities) that should be present on the first day of the advanced training program	
- Constructive alignment of curricula as a principle	
- Fostering of self-directed learning	
**Structured Curriculum Planning**<br>*Characteristics / Actions:*	
- Theory-based approach	
- During processes of fundamental change, project management should be adequately equipped and institutionally integrated	

**Curricular Requirements** (explicit in curriculum) (continuation)	**exists**
**Transparent Curricular Framework**<br>*Characteristics / Actions:*<br>Definition of:	
- Teaching formats	
- Hours, class times	
- Classrooms	
- Staff-student-ratio	
- Relationship between theory and practice, transparent objectives, resources for self-guided exercises (learning center/skills lab), reserved time for e-learning and blended learning, elective courses for individual specialization	
**Transparent Assessments**<br>*Characteristics / Actions:*	
- Transparent learning objectives (e.g. listed online)	
- Transparent examination procedures (registration deadlines, responsible people, objections and appeals procedures)	
- Balance between formative and summative assessment formats	
- Routine determination of (interim) qualifications (e.g. OSCE to assess readiness for medical clerkship)	
- Progress test in medicine (for intra- and inter-school comparisons)	
**Student Participation**<br>*Characteristics / Actions:*	
- Student participation at institutional level (medical school bodies, curriculum commissions, course design, course evaluations)	
- Inclusion of students in teaching (tutor programs / peer-assisted learning)	
**Individual Support**<br>*Characteristics / Actions:*	
- Individualized elective offerings and specialty programs (e.g. MD/ PhD)	
- Mentoring Programs	
- Student advising	
- Childcare	

**Qualification of Teaching Staff** [57–62]	**exists**
Characteristics / Actions:	
- Focus on recommendations for teacher qualification	
- Basic teacher qualification for all instructors	
- Modular structure	
- Medical teaching qualification regarding [the rules and regulations for] promotion and tenure	
- Coaching and mentoring programs for teaching staff	
- Fostering communities of practice	
- Program certification, e.g. *Medizin Didaktik Netzwerk* (MDN), *Certificate of Advanced Studies* (CAS; 10 ECTS), *Diploma of Advanced Studies* (DAS; 30 ECTS), or as a Master's degree program (60 ECTS, MME, etc.)	
- *Tenure track* positions (professorships)	
**Material and Personnel Resources** [33] [63–65]	**exists**
**Suitable and Sufficient Rooms**<br>*Characteristics / Actions:*	
- Rooms suitable for the selected teaching formats (e.g. for lab practicums, small-group instruction, learning center/skills lab)	
- Audio / visual equipment (e.g. audio response systems, practice phantoms and models)	
- Spaces for self-study (e.g. medical library, access to relevant literature and online training programs)	
- Teaching spaces and changing rooms in the hospital	
- Central classroom building / center for collaborative learning (if applicable with a dean of studies)	
- Central administration for optimal use of available rooms and spaces	
**Personnel Resources**<br>Characteristics / Actions:	
- Qualified teaching staff (existence of training programs, leave to prepare, teach and wrap-up courses)	
- Qualified academic personnel to develop the curriculum	
- Sufficient administrative and technical personnel	

**Time Constraints** [12], [27], [56], [66], [67]	**exists**
Characteristics / Actions:	
**Instructors**	
- Calculation of teaching duties including actual classroom time, course preparation and organization, development and adaptation of lesson plans, modules and overall curriculum; for exams: time for preparation, administration and grading	
**Students**	
- No overlapping of mandatory courses in terms of scheduling	
- Allowance for individual learning outside of the classroom sessions (preparation and follow-up, blended learning, exam preparation), as well as time for travel and breaks	
- Approximately 20 contact hours per weeks results in 50 – 60 hours of work per week and can be viewed as optional	
**Criteria for Successful Teaching and Learning Outcomes** [9], [18], [47]	**exists**
- Length of study	
- Exam scores, if applicable with subject-related information	
- Results of progress tests (cross-sectional and longitudinal)	
- Cross-medical school comparisons of OSCE scores (standardized stations)	
- Results of graduate surveys, e.g. career development analysis	
- Student satisfaction	
